# Characterization of C-S Lyase from *C. diphtheriae*: A Possible Target for New Antimicrobial Drugs

**DOI:** 10.1155/2013/701536

**Published:** 2013-09-11

**Authors:** Alessandra Astegno, Alejandro Giorgetti, Alessandra Allegrini, Barbara Cellini, Paola Dominici

**Affiliations:** ^1^Department of Biotechnology, University of Verona, 15 Strada Le Grazie, 37134 Verona, Italy; ^2^Department of Life Sciences and Reproduction, University of Verona, 8 Strada Le Grazie, 37134 Verona, Italy

## Abstract

The emergence of antibiotic resistance in microbial pathogens requires the identification of new antibacterial drugs. The biosynthesis of methionine is an attractive target because of its central importance in cellular metabolism. Moreover, most of the steps in methionine biosynthesis pathway are absent in mammals, lowering the probability of unwanted side effects. Herein, detailed biochemical characterization of one enzyme required for methionine biosynthesis, a pyridoxal-5′-phosphate (PLP-) dependent C-S lyase from *Corynebacterium diphtheriae*, a pathogenic bacterium that causes diphtheria, has been performed. We overexpressed the protein in *E. coli* and analyzed substrate specificity, pH dependence of steady state kinetic parameters, and ligand-induced spectral transitions of the protein. Structural comparison of the enzyme with cystalysin from *Treponema denticola* indicates a similarity in overall folding. We used site-directed mutagenesis to highlight the importance of active site residues Tyr55, Tyr114, and Arg351, analyzing the effects of amino acid replacement on catalytic properties of enzyme. Better understanding of the active site of *C. diphtheriae* C-S lyase and the determinants of substrate and reaction specificity from this work will facilitate the design of novel inhibitors as antibacterial therapeutics.

## 1. Introduction

The emergence of resistance to antibacterial agents is a pressing concern for human health. New drugs to combat this problem are therefore in great demand. Acquired bacterial resistance has caused several antibiotics to become useless or, at best, compromised in their ability to counteract bacterial infection [1]. The potential of amino acid biosynthesis as an antimicrobial target has been validated both chemically and biochemically [2]. Methionine represents a key amino acid in prokaryotes and it is an attractive antimicrobial target because of its important role in cell metabolism. Methionine, in the form of S-adenosylmethionine, is the methyl donor for a number of essential biochemical reactions. The biosynthesis of methionine is therefore of vital importance to microbial growth. This has been validated by the fact that several natural products, including 2-amino-5-hydroxy-4-oxopentanoic acid [3], azoxybacilin [4], and rhizocticin [5], target important enzymes for methionine biosynthesis and have antimicrobial properties. Moreover, most of the steps in the methionine biosynthesis pathway are unique to bacteria and plants. 

We have studied one of the enzymes required for methionine biosynthesis, namely, NCBI protein NP_940074, which has been annotated as a pyridoxal-5′-phosphate (PLP-) dependent C-S lyase from *Corynebacterium diphtheriae*, a pathogenic bacterium that causes diphtheria. The threat of diphtheria, even in countries with good coverage in childhood immunization programs by vaccination, has not disappeared and outbreaks in many European countries have revealed a phenomenon of decreasing immunity to diphtheria among adults [6]. The treatment of diphtheria patients is based on administration of antibiotics to eliminate the corynebacteria from the site of infection, thus stopping ongoing toxin production with or without additional treatment with specific immunoglobulins [7]. Thus, while diphtheria antitoxin (DAT) contributes to reducing the case fatality rates of infections due to this microorganism, it is unlikely, in the foreseeable future, that it will substantially reduce the world's consumption of antimicrobial agents. This highlights the importance of the development of new antibacterial drugs. 

C-S lyase catalyzes the *α*,*β*-elimination of sulfur-containing amino acids, such as L-cystathionine (L-Cth), to generate ammonia, pyruvate, and homocysteine, the penultimate step in methionine biosynthesis transsulfuration pathway. The crystal structure of *C. diphtheriae* C-S lyase has been determined by X-ray crystallography at 1.99 Å resolution (PDB code: 3FDB, Joint Center for Structural Genomics, http://www.jcsg.org/). According to its folding pattern, the enzyme belongs to the fold type I family of the PLP-dependent enzymes [8]. The commonly known fold type I PLP-dependent enzymes have an aromatic amino acid residue located at the *re *face of the PLP-Lys internal aldimine and stacking with the pyridine ring of PLP. The structure shows that a conserved binding site for PLP which is covalently linked to Lys222 has a stacking interaction with Tyr114 and H-bond interaction with Asn160, Asp188, and His191. There is one molecule in each asymmetric unit (PDB code: 3FDB). Although the crystal structure has been solved, no protein characterization and structure-based design studies have been reported to date. 

The availability of the protein in purified form has allowed us to obtain an insight into the biochemical properties of the enzyme by thorough characterization of its kinetic and spectral properties. With increasing resistance of bacteria to common antibiotics, C-S lyase appears to be an interesting novel target for the development of specific inhibitors against the pathogen *C. diphtheriae*.

## 2. Materials and Methods

### 2.1. Chemicals

PLP, L-cystathionine, L-cystine, L-cysteine, L-djenkolic acid, L-serine, aminoethyl-L-cysteine, O-acetyl-L-serine, L-homoserine, L-methionine, phenylhydrazine hydrochloride, NADH, pyruvate, rabbit muscle L-lactic dehydrogenase, 5,5′-dithiobis-(2-nitrobenzoic acid), isopropyl *β*-D-thiogalactoside, and the gel filtration molecular mass marker kit were obtained from Sigma. Chromatographic columns were from GE Healthcare, synthetic oligonucleotides were from Invitrogen, and the QuikChange site-directed mutagenesis kit was from Stratagene. pSpeedET-NP_940074 vector was obtained from DNASU/PSI:Biology-MR plasmid repository at the Biodesign Institute at Arizona State University [9, 10]. All other chemicals were the highest grade commercially available.

### 2.2. Enzyme Purification

Expression of the protein was carried out using the pSpeedET-NP_940074 vector by growing freshly transformed *E. coli* BL21(DE3) cells in LB medium. Cell cultures were grown at 37°C with vigorous shaking to an OD of 0.6 at 600 nm. The temperature was then lowered to 23°C and, after induction with 0.5 mM isopropyl-*β*-D-1-thiogalactopyranoside (IPTG), cells were grown for 16 h, harvested by centrifugation, resuspended in extraction buffer (20 mM sodium phosphate pH 7.5, 200 mM sodium chloride, and 10 mM imidazole), and lysed by sonication. The cell debris was removed by centrifugation (20,000 ×g for 30 min) and the supernatant was loaded onto an Ni-affinity column equilibrated with 20 mM sodium phosphate at pH 7.5, 200 mM sodium chloride, and 10 mM imidazole. The imidazole concentration was increased stepwise, first to 30 mM to remove nonspecifically bounded proteins, and then to 500 mM to elute the enzyme. Monomer concentration was determined from the calculated extinction coefficient (*ε*_280 nm_ = 65048 M^−1 ^cm^−1^; http://web.expasy.org/protparam/). PLP content of holoenzyme was determined by releasing the coenzyme in 0.1 M NaOH and by using *ε*_388 nm_ = 6600 M^−1 ^cm^−1^. The yield from a standard purification was approximately 25 mg/L culture. The protein was stable at −80°C for many months and freezing and thawing did not alter the activity of the isolated enzyme.

### 2.3. Site-Directed Mutagenesis

Point mutants were made on the wild type construct pSpeedET-NP_940074 using the QuikChange site-directed mutagenesis kit (Stratagene), following the manufacturer's protocols. For each mutant, two synthetic oligonucleotide primers were designed, each was complementary to the opposite strands of the plasmid and contained the desired mutation. The coding region of all mutated plasmids was verified by DNA sequencing. *E. coli* strain BL21(DE3) cells were transformed and used for expression. The conditions for expression and purification of the mutants were as described for the wild type enzyme.

### 2.4. Enzyme Activity Assays

Activity was measured at 30°C on a Jasco-V560 UV-Vis spectrophotometer. The assay buffer was composed of 50 mM MOPS, 50 mM bicine, 50 mM proline pH 9.0, and 20 *μ*M PLP. Reactions were initiated by the addition of 1 *μ*M enzyme. The activity of C-S lyase toward L-Cth was detected via two spectrophotometric assays by using the reaction of 5,5′-dithiobis-(2-nitrobenzoic acid) (DTNB) with the free thiol of the L-homocysteine product (*ε*_412 nm_ = 13600 M^−1^ cm^−1^) and by monitoring pyruvate formation with the coupling enzyme NADH-dependent lactate dehydrogenase (LDH) which reduces the pyruvate product to lactate, with the concomitant conversion of NADH to NAD^+^ (*ε*_340 nm_ = 6200 M^−1 ^cm^−1^). The LDH assay was also employed to monitor C-S lyase activity toward other substrates.

Initial velocity data obtained by varying substrate concentrations were fitted to the hyperbolic form of the Michaelis-Menten equation

(1) $$\begin{array}{c} \frac{v}{E_{t}}=\frac{k_{\text{cat}}S}{\left(K_{m}+S\right)}. \end{array}$$

In the case of deviations from hyperbolic kinetics, initial velocity data obtained by varying substrate concentrations were fitted to a nonhyperbolic curve to (2) that takes into account substrate inhibition, that is, binding of a second molecule of substrate to the ES complex to form an inactive ternary complex SES [11]:

(2) $$\begin{array}{c} \frac{v}{E_{t}}=\frac{k_{\text{cat}}}{1+\left(K_{m\;}/S\right)+\left(S/K_{i}\right)}, \end{array}$$

where *E*_*t*_ is the total enzyme concentration, *k*_cat_ is the maximum velocity, *S* is the substrate concentration, *K*_*m*_ is the apparent Michaelis-Menten constant, and *K*_*i*_ is the dissociation constant for the inhibitory SES ternary complex.

### 2.5. Evaluation of the pH Dependence

The pH dependence for C-S lyase towards L-Cth and L-cysteine was determined using both DTNB and LDH assays in 50 mM MOPS, 50 mM bicine, 50 mM proline, and 20 *μ*M PLP. Kinetic measurements were carried out between pH 7.8 and 10.3. The values for log⁡⁡*k*_cat_/*K*_*m*_ as a function of pH were fitted to the appropriate equations:

(3) $$\begin{array}{c} \log Y=\log\frac{C}{1+\left(H/K_{A}\right)}, \end{array}$$

(4) $$\begin{array}{c} \log Y=\log\frac{C}{1+\left(H/K_{A}\right)+\left(K_{B}/H\right)}, \end{array}$$

where *K*_*A*_ and *K*_*B*_ represent the ionization constants for enzyme or reactant functional group, *Y* is the value of the parameter observed as a function of pH, and *C* is the pH-independent value of *Y*. All data were fitted to the previous equations using the Origin8 program (OriginLab).

### 2.6. Spectroscopic Measurements

Absorption measurements were carried out on a Jasco-V560 UV-Vis spectrophotometer, using 10 *μ*M enzyme in a buffer solution containing 50 mM MOPS, 50 mM bicine, and 50 mM proline pH 8.5.

Fluorescence spectra were obtained with a Jasco FP8200 spectrofluorometer using 5 nm bandwidths on both sides at a protein concentration varying from 1 to 10 *μ*M in a solution containing 20 mM Bis-Tris propane pH 8.5. Spectra of blanks, that is of samples containing all component except the lyase, were taken immediately prior to the measurements of samples containing protein. Blank spectra were subtracted from spectra of samples containing enzyme.

CD measurements were carried out on a Jasco J-710 spectropolarimeter using 1 mg/mL enzyme in a buffer solution containing 50 mM Tris-HCl pH 8.5. For near-UV and visible wavelengths, three automatically averaged spectra were recorded in a cuvette with a 1 cm path length at a scan speed of 50 nm/min.

### 2.7. Preparation and Reconstitution of Apoenzyme

The coenzyme PLP was removed as a phenylhydrazone following the protocol in [12]. After the removal of the cofactor, C-S lyase exhibited no residual activity and no absorption peak at 413 nm. Titration of the apoenzyme with PLP was carried out at equilibrium by measuring the fluorescence emission at 500 nm (*λ*_exc_ = 413 nm) after incubation of apoprotein (5 *μ*M) with various concentrations of the cofactor (0–100 *μ*M) at 25°C for 15 min in 20 mM Bis-Tris propane pH 8.5. The increase in fluorescence emission at 500 nm was dependent on added PLP and the values were used to calculate the *K*_*d*_^PLP^ according to a tight-binding hypothesis (5):

(5) $$\begin{array}{c} F_{500}=\Delta F_{\max}\frac{e_{0}+l_{0}+K_{d}-\sqrt{\left[{\left(e_{0}+l_{0}+K_{d}\right)}^{2}-4e_{0}l_{0}\right]}}{2e_{0}}, \end{array}$$

where *e*_0_ and *l*_0_ are the total concentrations of C-S lyase subunit and PLP, respectively, *K*_*d*_ is the equilibrium dissociation constant for the encounter complex, and Δ*F*_max⁡_ represents the fluorescence change at 500 nm recorded in the presence of an excess of PLP over apoprotein. The reconstituted enzyme completely recovered its original activity.

### 2.8. Size Exclusion Chromatography

The molecular weight of recombinant enzyme was monitored by analytical size exclusion chromatography (Superdex 200 HR 10/30) using ÄKTA FPLC system (GE Healthcare). The chromatography was performed using 20 mM sodium phosphate buffer pH 7.2 and 150 mM NaCl as the mobile phase, at a flow rate of 0.1 mL/min. The calibration curve was determined with gel filtration molecular weight standards containing bovine thyroglobulin (669 kDa), apoferritin (443 kDa), *β*-amylase (200 kDa), alcohol dehydrogenase (150 kDa), bovine serum albumin (66 kDa), carbonic anhydrase (29 kDa), and cytochrome c (12.4 kDa). The curve was linear between 12.4 and 669 kDa.

### 2.9. Structural Similarity Analysis

The structure of the C-S lyase in complex with the PLP-aminoethoxyvinylglycine (AVG) was obtained by optimal superposition of the main chain of the C-S lyase (PDB code: 3FDB) and the structure of the cystalysin from *Treponema denticola* (PDB code: 1C7O) as in [13].

## 3. Results

Recombinant C-S lyase was purified as a His-tagged protein to greater than 99% purity as confirmed by SDS-PAGE; the size of the protein, calculated with a molecular size marker, was about 44 kDa that corresponds well to the molecular mass elucidated from the sequence information. As determined by gel filtration, an apparent molecular mass of 85 kDa was calculated for the native enzyme. Therefore, these results indicate that, as suggested by interface interaction, recombinant C-S lyase from *C. diphtheriae* is a homodimer.

### 3.1. Spectral Properties of Recombinant C-S Lyase

**Figure 1:**
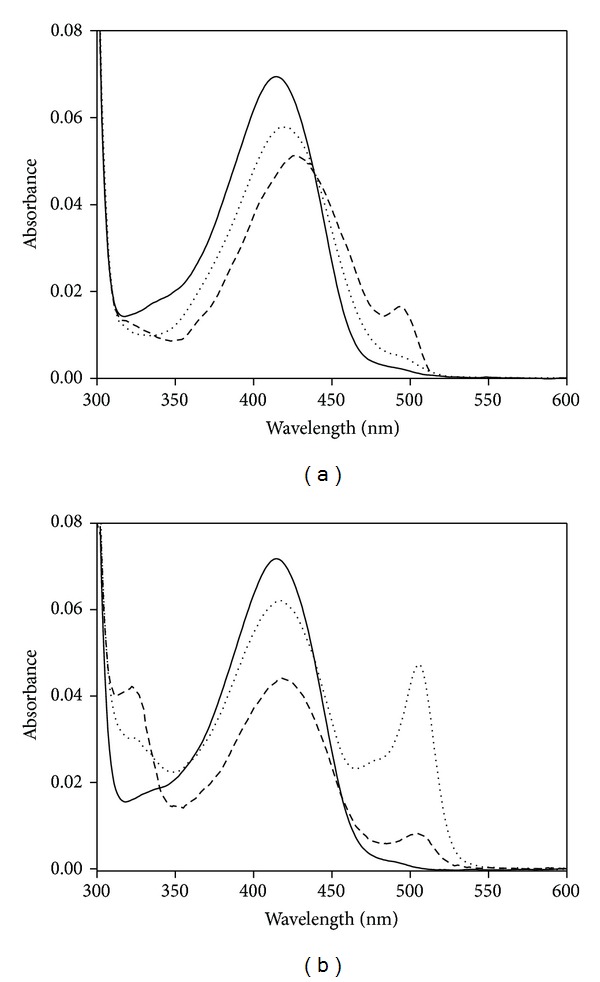
Absorption spectra of C-S lyase in the presence of substrates and substrate analogues. (a) Absorption spectra of 10 *μ*M C-S lyase in the absence (solid line) and presence of 100 mM L-serine (dashed line) and 100 mM O-acetyl-L-serine (dotted line). The spectra were subtracted from pyruvate absorbance. Pyruvate concentration was evaluated by a coupled LDH assay (see Section 2). (b) Absorption spectra of 10 *μ*M C-S lyase in the absence (solid line) and presence of 450 mM L-homoserine (dotted line) and 150 mM L-methionine (dashed line). All spectra were recorded in a solution containing 50 mM MOPS, 50 mM bicine, and 50 mM proline pH 8.5.

The visible absorption spectrum of the native enzyme is dominated by a band centered around 413 nm (Figure 1). This peak indicates that the predominant tautomer of the internal aldimine is the ketoenamine, and the imine nitrogen of the cofactor is protonated allowing the formation of an intramolecular hydrogen bond with the 3′-oxygen atom of PLP and the conjugation of the *π* system of the imine with the pyridine ring [14, 15]. 

The intensity of the band did not change in the pH range from 6 to 11, indicating a p*K*_*a*_ of the internal aldimine higher than 11. The observed ratio *A*_280 nm_/*A*_413 nm_ was ~8. The stoichiometric ratio of PLP to enzyme was calculated from the *A*_388 nm_ of the free cofactor, which was released upon incubation of the native holoenzyme in 0.1 M NaOH. By this method, a PLP content of 2 mol/mol of the native dimer was found.

**Figure 2:**
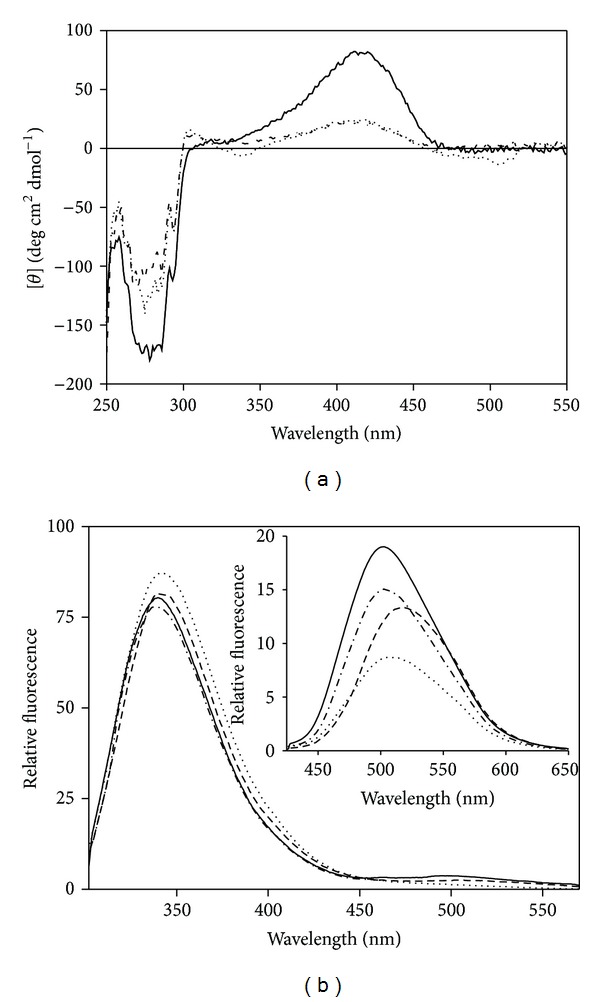
CD and fluorescence emission spectra of C-S lyase in the presence of substrates and substrate analogues. (a) CD spectra of enzyme (solid line) in the presence of 450 mM L-homoserine (dotted line) and 150 mM L-methionine (dashed line) in 50 mM Tris-HCl pH 8.5. (b) Fluorescence emission spectra of 1 *μ*M enzyme (solid line) in the presence of 150 mM L-methionine (dotted line), 100 mM L-serine (dashed line), and 100 mM O-acetyl-L-serine (dash dotted line) upon excitation at 295 nm. *Inset:* emission spectra of 10 *μ*M enzyme (solid line) in the presence of 100 mM O-acetyl-L-serine (dash dotted line), 100 mM L-serine (dashed line), and 150 mM L-methionine (dotted line) upon excitation at 418 nm. All spectra are recorded in 20 mM Bis-Tris-propane pH 8.5.

As shown in Figure 2(a), the CD spectrum of the enzyme displays a positive dichroic band at 415 nm whose intensity does not change with pH. The near-UV spectrum is characterized by pronounced negative dichroic bands in the aromatic region at 260–296 nm, which would indicate the asymmetry of certain aromatic amino acids, most likely associated with the active site (Figure 2(a)).

The fluorescence emission spectra of C-S lyase recorded for the direct excitation at 295 nm of Trp residues exhibit a pronounced peak centered around 340 nm and a much lower intensity peak at around 500 nm (Figure 2(b)). The peak at 340 nm can be attributed to direct emission from Trp residues, while the peak centered at 505 nm can be attributed to emission from PLP, resulting from an intramolecular energy-transfer process from the excited Trp residues to the cofactor [16, 17]. Direct excitation of the C-S lyase ketoenamine tautomer of PLP at 413 nm gives a single emission peak centered at 502 nm (Figure 2(b), inset), which is typical for emission from Schiff bases of PLP in a neutral, aqueous environment [18, 19]. None of the fluorescence emission properties of the enzyme is affected by pH in the range from 5.7 to 10.2. 

At pH 8.5 in 20 mM Bis-Tris propane, the excitation spectrum for the longer-wavelength emission of C-S lyase measured at 502 nm shows two bands. The peak of the shorter wavelength excitation band is located at 279 nm, and the peak of the longer-wavelength excitation band is located at 413 nm (data not shown).

The emission spectrum obtained for apo C-S lyase, when excited at 295 nm, exhibits a single peak centered at 345 nm, with a 4-fold increase in emission intensity and a 2 nm red shift compared to the holoenzyme (data not shown). The apoenzyme has no residual activity and does not exhibit either absorbance bands in the visible region or PLP emission fluorescence. Titration of apo C-S lyase with PLP resulted in an increase in the fluorescence emission at 500 nm; the values were plotted against PLP concentrations and used to calculate the *K*_*d*_^PLP^ value for the enzyme by a theoretical curve fit of the experimental data using (5). The obtained *K*_*d*_^PLP^ value was 0.09 ± 0.03 *μ*M, indicating a tight binding of PLP to the enzyme. 

### 3.2. Steady-State Kinetic Studies

Several sulfur- and nonsulfur containing amino acids were utilized as potential substrates for the enzyme. The enzyme showed maximum activity at pH 9 and 30°C, as determined in 50 mM MOPS, 50 mM bicine, and 50 mM proline.  Table 1 shows that the enzyme has a relatively broad substrate specificity. L-Cth, L-djenkolate, and aminoethyl-L-cysteine were the most effective substrates. Cystine and L-cysteine, to a lesser extent, also acted as substrates. O-acetyl-L-serine and L-serine proved to be very poor substrates, whereas no activity could be detected with L-homocysteine, L-homoserine, or L-methionine. These latter inhibited, although to different extents, C-S lyase activity (data not shown), thus indicating their binding to the enzyme. The recombinant C-S lyase obeyed Michaelis-Menten kinetics with all substrates examined except for L-cysteine, for which substrate inhibition was observed. By using (2) (describing substrate inhibition, [11, 20]), initial velocity data obtained by varying L-cysteine concentrations fit a nonhyperbolic curve to yield *K*_*m*_ and *K*_*i*_ values of 0.95 ± 0.18 and 3.98 ± 0.66 mM, respectively.

### 3.3. pH Dependence of Kinetic Parameters for C-S Lyase

**Figure 3:**
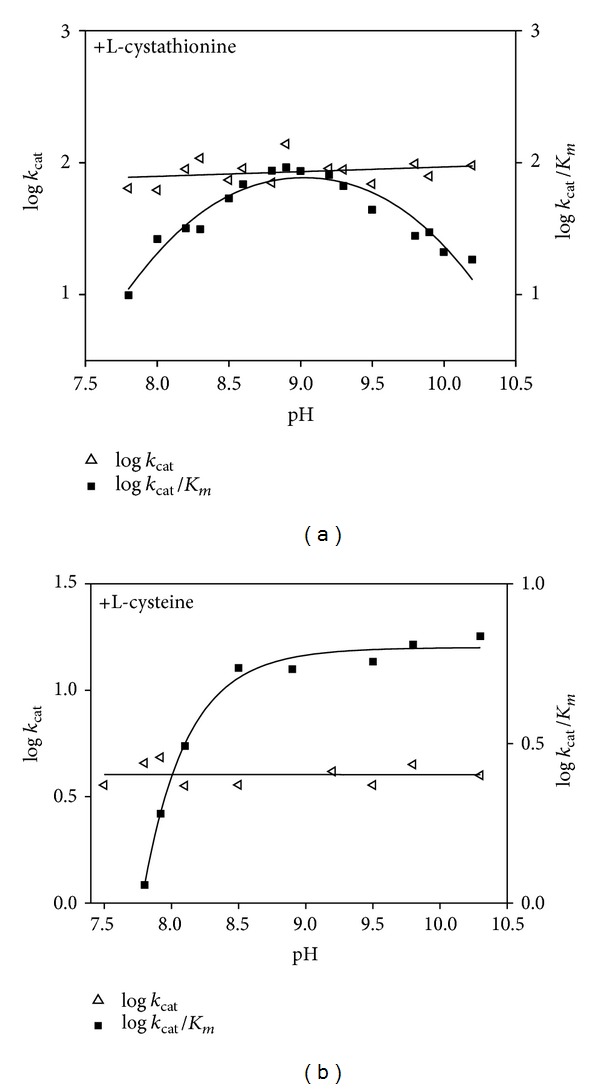
pH dependence of the kinetic parameters for C-S lyase reaction with L-Cth and L-cysteine. log⁡⁡*k*_cat_ profile and log *k*_cat_/*K*_*m*_ profile for L-Cth (a) and L-cysteine (b). The points shown are experimentally determined values, while the curves are from fits to the data using (3) for log⁡⁡*k*_cat_/*K*_*m*_ for L-cysteine and (4) for log⁡⁡*k*_cat_/*K*_*m*_ for L-Cth.

The pH dependence of the kinetic parameters for C-S lyase toward L-Cth and L-cysteine was determined, and the results are shown in Figures 3(a) and 3(b). log⁡⁡*k*_cat_ is pH independent from 7.8 to 10.2 for both substrates. The log⁡⁡*k*_cat_/*K*_*m*_—pH profile for L-Cth is bell shaped, consistent with the involvement of two ionizable groups, one with an apparent p*K*_*a*_ of 8.05 ± 0.07 and the other with an apparent p*K*_*a*_ of 9.90 ± 0.07 (Figure 3(a)). The log⁡⁡*k*_cat_/*K*_*m*_—pH profile for L-cysteine exhibited a single p*K*_*a*_ of 8.26 ± 0.01 for a group that must be deprotonated for activity (Figure 3(b)). 

### 3.4. Absorption, Fluorescence, and CD Spectral Changes of C-S Lyase with Substrates and Substrate Analogues

Except for L-serine and O-acetyl-L-serine, the rate of reaction for the substrates tested (Table 1) was very high, and reaction of the enzyme with these substrates cannot be studied by conventional spectroscopy.

Addition of O-acetyl-L-serine to C-S lyase at pH 8.5 produces an immediate shift from 413 to 420 nm (Figure 1(a)). This shift is consistent with the conversion of internal to external aldimine (Figure 1(a)). A broad increase in the absorbance at wavelengths shorter than 350 nm hampers the accurate identification of specific bands and is likely due to the accumulation of reaction products, that is, pyruvate, which absorbs at 318 nm [21]. After subtraction of the contribution of pyruvate absorbance to the absorbance spectra of enzyme, the external aldimine is the only evident intermediate during steady-state conditions. On the other hand, binding of L-serine at pH 8.5 to the enzyme results in mixtures of external aldimine and quinonoid species absorbing at 428 and 495 nm, respectively (Figure 1(a)).

Binding of the substrate analogues L-methionine or L-homoserine at pH 8.5 to the enzyme results in mixtures of external aldimine (*λ*_max⁡_ = 418 nm) and quinonoid species (*λ*_max⁡_ = 505 nm). This equilibrium is attained immediately upon mixing (Figure 1(b)). 

CD spectra of the PLP cofactor were obtained in the presence of L-methionine and L-homoserine (Figure 2(a)). The spectrum for unliganded enzyme exhibits a positive Cotton effect centered on the visible absorption band of the cofactor, as it has been found for many PLP enzymes including cystalysin [12] and O-acetylserine sulfhydrylase [22] which catalyze reactions very similar to that catalyzed by C-S lyase. In the presence of saturating concentrations of L-methionine and L-homoserine, a positive Cotton effect is still evident, but with only 30% ellipticity relative to enzyme alone. The presence of L-homoserine gives a small negative dichroic signal centered around the visible absorption band of the quinonoid intermediate at 502 nm, and a modest negative dichroic band at 337 nm, mimicking what is observed in the visible spectrum. 

Differences were also seen between the unliganded and liganded enzymes in the near-UV CD spectrum. Upon addition of ligands, a decrease of the negative dichroic signals in the Tyr spectral region was observed, which was more pronounced for L-methionine. Both these ligands have a pronounced effect on Trp residue(s) whose fine structure between 293 and 305 nm is clearly affected (Figure 2(a)).

Upon excitation at 295 nm, in the presence of L-serine, the cofactor emission band of C-S lyase exhibits a decrease in intensity compared to the internal aldimine emission spectrum (Figure 2(b)), while the Trp emission band is red shifted to 344 nm, maintaining almost the same intensity. The decrease in the emission band of the ketoenamine tautomer is confirmed by the direct excitation of the ketoenamine tautomer at 425 nm, which shows a decrease and a red shift to 517 nm compared to the emission of the internal aldimine (Figure 2(b), inset). When the enzyme was excited at 418 nm in the presence of O-acetyl-L-serine or L-methionine, the emission spectrum exhibited a quenching of the fluorescence emission at 500 nm, concomitant with a modest red shift to 504 or 506 nm, respectively (Figure 2(b), inset). 

### 3.5. Effects of Arg351, Tyr114, and Tyr55 Mutation on the Enzymatic Activity of C-S Lyase

**Figure 4:**
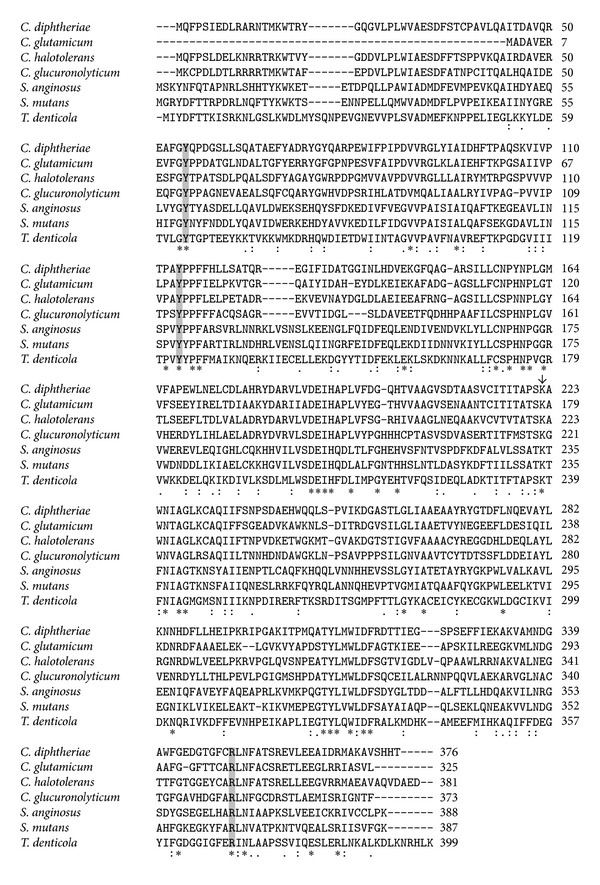
Sequence alignment of C-S lyases. The target residues for mutational analysis are highlighted in gray. The arrow above the sequences indicates the PLP-binding lysine. The C-S lyases used in this alignment (UniProt accession numbers) and their sources are Q6NFZ9, *Corynebacterium diphtheriae* NCTC 13129; Q46061, *Corynebacterium glutamicum*; M1NUD7,* Corynebacterium halotolerans*; C0VUP4, *Corynebacterium glucuronolyticum*; A6BMJ3, *Streptococcus anginosus* IMU102; Q8DST5, *Streptococcus mutans*; Q56257, *Treponema denticola* ATCC 3540. All sequence alignments were carried out using the ClustalW program.

**Figure 5:**
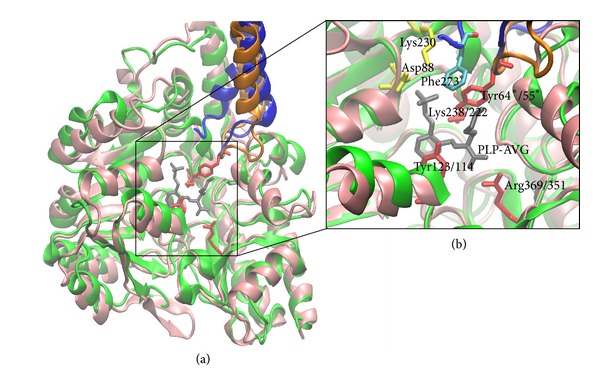
Superimposed structures of C-S lyase from *Corynebacterium diphtheriae *and cystalysin from *Treponema denticola*. (a) A subunit of C-S lyase (green) with a portion of chain B (orange) is superimposed with chains A and B of cystalysin (in pink and blue, resp.). (b) A close-up of the active site with the AVG inhibitor (in gray) is shown. Residues conserved in both proteins are shown in red. Phe273 is presented only in the chain B of cystalysin and is depicted in cyan, while residues Lys230 and Asp88 are presented only in C-S lyase and are shown in yellow. Residue numbers are indicated as cystalysin/C-S lyase. An asterisk (*) indicates that the residues are present only in chain B.

To identify the residues involved in catalysis and/or substrate binding, we aligned the amino acid sequences of seven bacterial C-S lyases (Figure 4). The C-S lyases share similar sequences and exhibit identities to C-S lyase from *C. diphtheriae* ranging from 27% (cystalysin from *T. denticola*) to 54% (C-S lyase from *C. glutamicum*). The alignment indicates that 40 amino acids are strictly conserved, and structural comparison of C-S lyase with cystalysin indicates similarity in overall fold as well as in active site residues (Figure 5). Of the 20 residues bordering the active site and the cofactor, we focused on three amino acids that are invariant among all the enzymes of the transsulfuration pathway. The conserved tyrosines (Tyr55* and Tyr114) and arginine (Arg351), located at the active site, were selected for point mutation based on structural analysis (Figure 5(b)). The effect of mutation on enzyme activity was examined (Table 2). Mutation of Tyr55 to Phe dramatically reduced enzymatic activity (*k*_cat_ = 0.45 s^−1^); this Tyr is located on the dimer interface, and its side chain directly interacts with the PLP of the other subunit. Mutant R351A reduced the activity below detectable levels. On the other hand, the Y114F mutant retained 1.3% of the turnover rate of wild type enzyme. Although Tyr114 and Arg351 are positioned near the dimer interface, there are no direct interactions with any residues of the other subunit.

### 3.6. Structural Similarity Analysis

Guided by structural superimposition and structural similarity analysis, interesting differences were found between cystalysin from *T. denticola *and C-S lyase from *C. diphtheriae *in the AVG adduct. Although the root mean square deviations (RMSD) between both structures are small, that is, 1.5 Å, there are important differences in the residues present in the binding cavity. Indeed, while in the cystalysin-AVG complex hydrophobic interactions are formed with Phe273 (cyan in Figure 5(b)), this residue is not conserved in C-S lyase, thus offering a larger binding cavity. On the other hand, there are two residues present in C-S lyase that are not conserved in cystalysin from *T. denticola*, that is, Asp88 and Lys230 (yellow in Figure 5(b)). Both these differences may dramatically affect the physicochemical properties of the binding cavity and allow the design of specific inhibitors. 

## 4. Discussion

The enzymes of the bacterial transsulfuration pathway, cystathionine *γ*-synthase and cystathionine *β*-lyase, are attractive targets for the development of novel antimicrobial compounds because this pathway is unique to plants and bacteria. Recent studies probing the active sites of these enzymes are providing information that will guide the design of novel therapeutics [2, 23]. As members of the *γ*-subfamily of fold-type I of PLP-dependent enzymes, they also represent a useful model system for studies exploring the molecular mechanisms that underlie specificity among enzymes dependent on this catalytically versatile cofactor. A thorough understanding of the mechanisms controlling substrate and reaction specificity is a necessary step to enable the engineering of PLP-dependent enzymes for biotechnological applications [24].

The structure of *C. diphtheriae* C-S lyase has been solved at 1.99 Å resolution (PDB code: 3FDB). We isolated the recombinant enzyme as a His-tagged protein at high yield and examined the spectral properties and steady state kinetic parameters of the purified enzyme, in addition to structural similarity analysis with cystalysin.

The internal aldimine absorbance spectra of C-S lyase exhibit a band centered at 413 nm (Figure 1(a)) which indicates that the predominant tautomer of the internal aldimine is the ketoenamine and that the imine nitrogen of the cofactor is protonated. This band is associated with a positive Cotton effect and gives rise to an emission maximum at about 500 nm. The enzyme spectrum is independent of pH from 6 to 11, indicating that the p*K*_*a*_ of the internal aldimine is higher than pH 11. In the structurally related enzyme cystalysin, a band at 320 nm was attributed to a substituted aldimine, a species stabilized with respect to the ketoenamine by alkaline pH [12]. Both cystalysin and C-S lyase are catalytically more competent at alkaline pH, where a remarkable portion of the coenzyme in cystalysin exists as inactive aldimine structure with a nucleophilic group. Interestingly, the spectrum of the *C. diphtheriae* is pH independent; thus, this nucleophilic group is either missing or has a different location.

The apoenzyme showed no absorption in the visible range and no emission fluorescence due to PLP. When the intrinsic fluorescence emissions of holo- and apoenzyme were compared, it was evident that a substantial fraction (about 70%) of the Trp emission in the apoenzyme is quenched in the holoenzyme. Such quenching is concomitant with the appearance of the 500 nm emission indicative of energy transfer from Trp residue(s) to the internal Schiff base. The interaction between the apoenzyme and PLP is characterized by a dissociation constant of 0.09 *μ*M, which is indicative of very tight binding of the cofactor to the enzyme.

Conversion to the external aldimine upon addition of substrate or substrate analog is expected to involve a conformational change that causes, among other things, the exclusion of water molecules from the active site. Formation of the external aldimine with L-serine is accompanied by a bathochromic shift of the ketoenamine tautomer absorption band (15 nm; Figure 1(a)) compared to the same species with O-acetyl-L-serine, L-methionine, or L-homoserine (about 6 nm). This finding suggests a pronounced increase in the polarity of the microenvironment in the active site, likely due to a repositioning of charged or polar residues around the cofactor. Binding of L-serine to the enzyme results in mixtures of external aldimine and quinonoid species (Figure 1(a)). *α*,*β*-Elimination mechanisms generally proceed via a quinonoid intermediate, in which the carbanion formed upon abstraction of the C*α*-proton is stabilized by delocalization into the pyridinium ring of PLP. This transient intermediate with *λ*_max⁡_ ~ 450–500 nm is detectable when the elimination of the leaving group is slow, as might be expected for the hydroxyl group of L-serine [25]. The steady-state spectrum represents an intermediate whose formation is rate limiting or represents the sum of more than one intermediate if multiple steps are partially rate limiting [26]. Therefore, the presence of both the external aldimine and the substrate quinonoid in the steady-state spectrum of C-S lyase with L-serine indicates that the formation of these intermediates may be partially rate determining. In contrast, a quinonoid intermediate is not observed for the elimination of acetate from O-acetyl-L-serine as the acetate group of O-acetyl-L-serine does not require protonation and is a better leaving group than the hydroxyl group of the L-serine. Although L-methionine and L-homoserine bind to C-S lyase in an unproductive manner, they nonetheless give equilibrating mixtures of external aldimine and quinonoid species (Figure 1(b)). This implies that L-methionine or L-homoserine stops the reaction at the step involving the quinonoid intermediate. The effects of all amino acids tested on the CD and fluorescence spectra of C-S lyase consist, albeit to different extents, in a modest enhancement of intrinsic Trp fluorescence, quenching and red shift of the long wavelength PLP emission, and a change in the dissymmetry of bound cofactor and aromatic amino acids located near the active site (Figures 2(a) and 2(b)). Taken together, in terms of local perturbations these data can be interpreted as changes in the orientation of the PLP and in the relative orientations of an aromatic residue(s) and of cofactor upon binding of ligand.

The *k*_cat_ profile for the *α*,*β*-elimination reaction of L-Cth or L-cysteine is pH independent. The *k*_cat_/*K*_*m*_ versus pH profile for L-Cth is bell-shaped, with p*K*_*a*_ values of 8.05 ± 0.07 (p*K*_*a*1_ acidic limb) and 9.90 ± 0.07 (p*K*_*a*2_, basic limb). The *k*_cat_/*K*_*m*_^L-Cth^-pH profile decreased on the acid and basic sides with limiting slopes of 1 and −1, indicating that the ionization of the two groups is relevant to the enzymatic reaction, with one being protonated and the other unprotonated (Figure 3(a)).

The pH independence of the internal aldimine of C-S lyase indicates that this group cannot be assigned to one of those that titrates in the pH-activity profiles. The p*K*_*a*_ values of the R-NH_2_ groups of L-Cth were determined by direct titration with NaOH as 8.51 ± 0.02 and 9.60 ± 0.02 at 25°C, which are within experimental error of those determined for the acidic and basic limb of the *k*_cat_/*K*_*m*_^L-Cth^ versus pH plot.

The basic limb (p*K*_*a*2_) of the *k*_cat_/*K*_*m*_^L-Cth^ versus pH profile was assigned to the p*K*_*a*_ of the R-amino group of L-Cth, since p*K*_*a*2_ was not present in the *k*_cat_/*K*_*m*_^L-cys^ profile (Figure 3(b)). The acidic limb (p*K*_*a*1_) can be assigned to the *α*-amino group of L-Cth (p*K*_*a*_ = 8.54), indicating that the substrate must bind in the deprotonated state. Alternatively, the p*K*_*a*1_ could reflect ionization of an enzyme group other than the imino moiety of the internal aldimine.

**Scheme 1:**
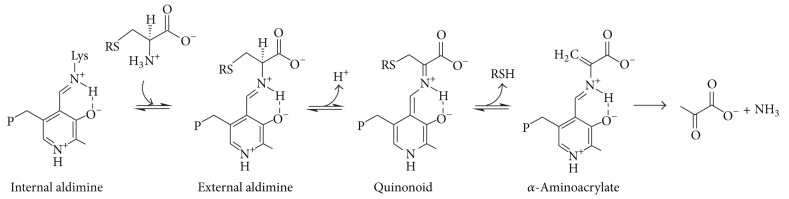
Key intermediate steps of *α*,*β*-elimination reaction.

It is generally accepted that PLP-dependent *β*-eliminases, including *β*C-S lyases, have three key intermediate steps [8] (Scheme 1). Plausible catalytic mechanisms for the formation of intermediates have been reported for several *β*C-S lyases on the basis of structural and spectroscopic information [12, 27–30]. Contrary to their common intermediates, the quaternary structures (dimer and tetramer) and the active site residues of the *β*C-S lyase enzymes are somewhat different, possibly depending on their substrate specificity. When studying the *α*,*β*-elimination reactions of sulfur containing amino acids in bacteria, determination of enzyme substrate specificity is crucial since it allows distinction between true cystathionine *β*-lyase activities involved in the synthesis of methionine and other C-S lyase activities. Thus, cystathionine *β*-lyase supports the cleavage of either L-Cth or djenkolate at high levels and shows low activity towards L-cystine. Conversely, C-S lyases are characterized by marginal cleavage of L-Cth and efficient synthesis of cysteine persulfide from L-cysteine [31, 32]. 

To define the substrate specificity of C-S lyase from *C. diphtheriae*, we examined the reaction of the enzyme with sulfur- and nonsulfur containing amino acids as well as with disulfidic amino acids (Table 1). Among the substrates tested, only L-cysteine exhibits substrate inhibition, similar to *T. denticola *cystalysin [12], C-DES from *Synechocystis *[33], **β*-*cystathionase [31], and D-cysteine desulfhydrase from *E. coli* [34].

Recombinant C-S lyase from* C. diphtheriae* showed a relatively broad substrate specificity toward sulfur-containing amino acids, as previously observed for the enzyme from *A. thaliana* [35], *E*. *coli *[31], *B*. *avium* [36], and *S*. *typhimurium *[37]. For all these enzymes, the highest rates of *β*-cleavage activity were obtained with L-Cth and djenkolate, whereas L-cystine and L-cysteine were poor substrates. In the case of L-cystine and L-cysteine, compared with L-Cth, substrate selectivity does not appear to derive from substrate binding, since *K*_*m*_ values are similar, but rather from chemical steps that follow the binding (Table 1). Finally, no activity could be detected for the enzyme when either L-homocysteine or L-methionine was used as substrate (*γ*C-S linkages).

The present observations are in agreement with previous analysis of the substrate specificity of cystathionine *β*-lyase from *A. thaliana* [35] which showed the following: (1) all active substrates contain a C3 L-*α*-amino acid moiety; (2) the nature of the sulfur-containing bridge (thioether, thioacetal, or disulfide) influences the rate of the *α*,*β*-elimination process; and (3) the length of the second moiety, and therefore the spatial separation of the two carboxyl groups of the substrate, has a critical role in the efficiency of cleavage. In light of these findings, *C. diphtheriae* should more properly be considered as a cystathionine *β*-lyase, rather than a C-S lyase, as annotated (NCBI protein NP_940074). Interestingly, although both *C. diphtheriae *C-S lyase and cystalysin belong to fold type I, and share similar sequences (Figure 4) and structural similarity in overall fold and active site residues (Figure 5), they do not display desulfhydrase activity with similar substrate specificity. Cystalysin, in fact, displays a strong substrate preference for L-djenkolic acid and L-cystine over L-cysteine and, among sulfur-containing amino acids, the lowest **β**-cleavage activity was obtained with L-Cth [12]; its substrate specificity is similar to that of L-cysteine/cysteine C-S lyase (C-DES) from *Synechocystis*. 

Based on structural and kinetic studies, a postulated catalytic mechanism with the intermediates has been proposed for cystalysin [38, 39] and *Streptococcus anginosus* *β*C-S lyase (Lcd) [40], the enzymes with the highest sequence identity to *C. diphtheriae* C-S lyase (Figure 5(a)) among structurally and enzymatically characterized *β*C-S lyases. The structural information on the intermediate complexes enabled us to select conserved residues for site-directed mutagenesis. Mutational analysis demonstrated the important roles of several active site residues, namely, Tyr55, Tyr114, and Arg351 (Figure 5(a)). In particular, Tyr114 recognizes the S *γ*-atom (or O *γ*-atom) of the substrate by the phenolic hydroxyl group and fixes PLP at the proper position by a stacking interaction throughout the *α*,*β*-elimination reaction [27, 38].

Mutational analysis further revealed that Y114F retained only 1.3% of wt enzymatic activity (Table 2). On the basis of kinetic studies, Cellini et al. [38] concluded that Tyr64 of cystalysin (Tyr55 of *C. diphtheriae* C-S lyase) plays a role in the properly orienting Lys238 (Lys222). In agreement with structural observations, substitution of Tyr55 to Phe reduced the catalytic activity to 0.5% (Table 2), suggesting the importance of the phenolic hydroxyl group of this residue. In both Lcd external aldimine and *α*-aminoacrylate forms, the guanidino group of Arg365 formed a salt bridge with the carboxyl moiety of the amino acid substrate bound to PLP [40]. This Arg is widely conserved among type I PLP-dependent enzymes and is supposed to act as a general *α*-carboxylate docking site [41]. A similar salt bridge has been observed between the corresponding Arg and the carboxyl moiety of the coenzyme bound substrate in other PLP-dependent *β*-eliminases [28, 42] suggesting a common role of the Arg for substrate binding. Substitution of Arg351 with Ala notably reduced catalytic activity toward L-Cth to below a detectable level (Table 2).

Although the residues involved in PLP and substrate binding are fully conserved, structural similarity analysis between cystalysin from *T. denticola *and C-S lyase from *C. diphtheriae *in the AVG adduct revealed subtle differences in the binding cavity. These involve just three amino acids, that is, two charged residues present in C-S lyase and not in cystalysin and *vice versa*, a Phe residue present in cystalysin and not in C-S lyase. Indeed, the availability of two charged residues and the lack of bulky hydrophobic one will dramatically affect the physicochemical properties of the binding cavity of C-S lyase. These elements can thus be exploited for rational design of novel and species specific inhibitors to be used in antibacterial therapeutics.

## Figures and Tables

**Table 1:** Kinetic parameters for substrates in 50 mM MOPS, 50 mM bicine, and 50 mM proline pH 9.0 at 30°C.

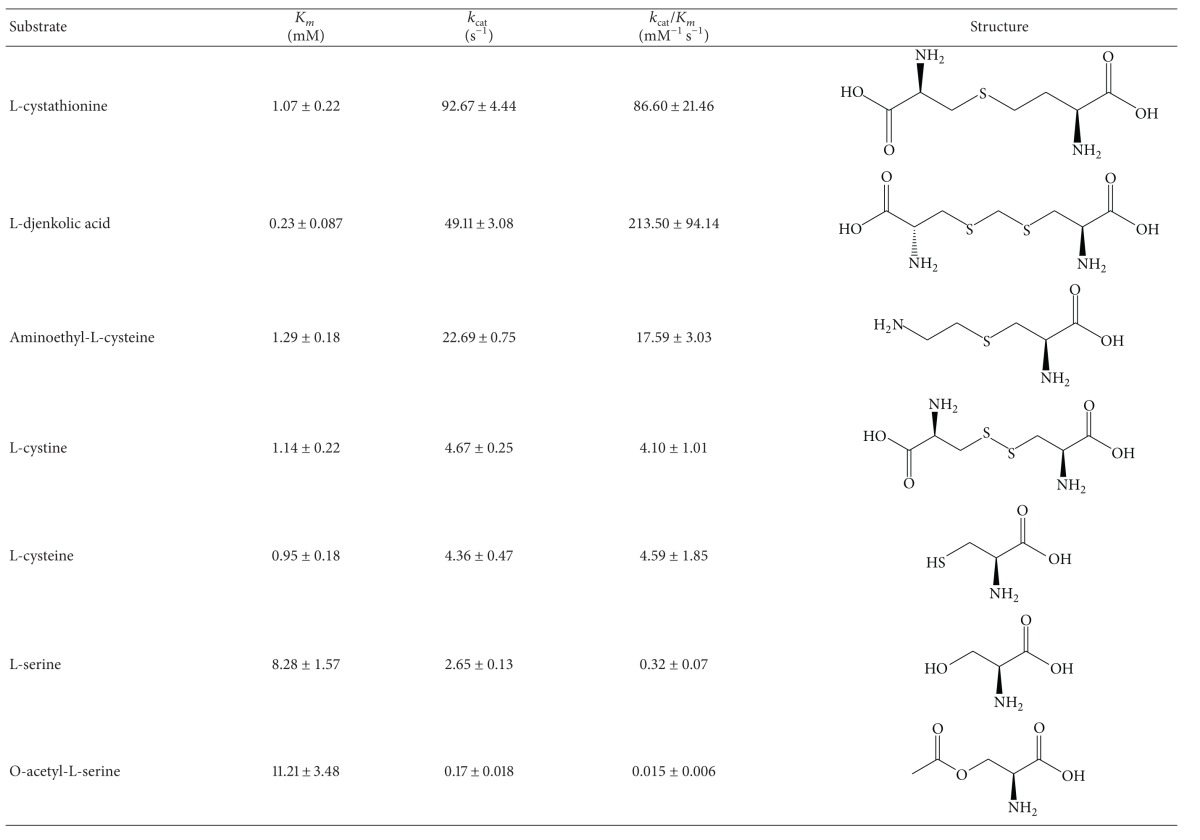

**Table 2:** Kinetic parameters for C-S lyase wt and mutants using L-Cth in 50 mM MOPS, and 50 mM bicine, 50 mM proline pH 9.0 at 30°C.

Protein	*K*_*m*_ (mM)	*k*_ cat_ (s^−1^)	Relative activity (%)
wt	1.07 ± 0.22	92.67 ± 4.44	100
Y114F	0.20 ± 0.01	1.20 ± 0.02	1.3
Y55F	0.62 ± 0.21	0.45 ± 0.01	0.5
R351A	n.d.*	n.d.*	—

*n.d.: not detected.

## Abbreviations

PLP: Pyridoxal 5′-phosphate
DTNB: 5,5′-dithiobis-2-nitrobenzoate
LDH: L-lactic dehydrogenase
L-Cth: L-cystathionine
AVG: L-aminoethoxyvinylglycine
*K*_*d*_^PLP^: Equilibrium dissociation constant for PLP
CD: Circular dichroism
PDB: Protein Data Bank
*k*_cat_: Turnover rate
*K*_*m*_: Michaelis constant.

## References

[1] Coates A, Hu Y, Bax R, Page C (2002). The future challenges facing the development of new antimicrobial drugs. *Nature Reviews Drug Discovery*.

[2] Ejim LJ, Blanchard JE, Koteva KP (2007). Inhibitors of bacterial cystathionine *β*-lyase: leads for new antimicrobial agents and probes of enzyme structure and function. *Journal of Medicinal Chemistry*.

[3] Jacques SL, Mirza IA, Ejim L (2003). Enzyme-assisted suicide: molecular basis for the antifungal activity of 5-hydroxy-4-oxonorvaline by potent inhibition of homoserine dehydrogenase. *Chemistry and Biology*.

[4] Aoki Y, Yamamoto M, Hosseini-Mazinani SM, Koshikawa N, Sugimoto K, Arisawa M (1996). Antifungal azoxybacilin exhibits activity by inhibiting gene expression of sulfite reductase. *Antimicrobial Agents and Chemotherapy*.

[5] Kugler M, Loeffler W, Rapp C, Kern A, Jung G (1990). Rhizocticin A, an antifungal phosphono-oligopeptide of *Bacillus subtilis* ATCC 6633: biological properties. *Archives of Microbiology*.

[6] Galazka A (2000). The changing epidemiology of diphtheria in the vaccine era. *Journal of Infectious Diseases*.

[7] Wagner KS, Stickings P, White JM (2009). A review of the international issues surrounding the availability and demand for diphtheria antitoxin for therapeutic use. *Vaccine*.

[8] Schneider G, Käck H, Lindqvist Y (2000). The manifold of vitamin B6 dependent enzymes. *Structure*.

[9] Cormier CY, Mohr SE, Zuo D (2010). Protein structure initiative material repository: an open shared public resource of structural genomics plasmids for the biological community. *Nucleic Acids Research*.

[10] Cormier CY, Park JG, Fiacco M (2011). PSI:Biology-materials repository: a biologist’s resource for protein expression plasmids. *Journal of Structural and Functional Genomics*.

[11] Copeland RA (2002). Kinetics of single-substrate enzyme reactions. *Enzymes: A Practical Introduction to Structure, Mechanism, and Data Analysis*.

[12] Bertoldi M, Cellini B, Clausen T, Voltattorni CB (2002). Spectroscopic and kinetic analyses reveal the pyridoxal 5′-phosphate binding mode and the catalytic features of *Treponema denticola* cystalysin. *Biochemistry*.

[13] Raimondo D, Giorgetti A, Bernassola F, Melino G, Tramontano A (2008). Modelling and molecular dynamics of the interaction between the E3 ubiquitin ligase Itch and the E2 UbcH7. *Biochemical Pharmacology*.

[14] Cook PF, Wedding RT (1976). A reaction mechanism from steady state kinetic studies for O acetylserine sulfhydrylase from *Salmonella typhimurium* LT-2. *The Journal of Biological Chemistry*.

[15] Becker MA, Kredich NM, Tomkins GM (1969). The purification and characterization of O-acetylserine sulfhydrylase-A from *Salmonella typhimurium*. *The Journal of Biological Chemistry*.

[16] Strambini GB, Cioni P, Peracchi A, Mozzarelli A (1992). Characterization of tryptophan and coenzyme luminescence in tryptophan synthase from *Salmonella typhimurium*. *Biochemistry*.

[17] McClure GD, Cook PF (1994). Product binding to the *α*-carboxyl subsite results in a conformational change at the active site of O-acetylserine sulfhydrylase-A: evidence from fluorescence spectroscopy. *Biochemistry*.

[18] Arrio-Dupont M (1970). Fluorescence study of Schiff bases of pyridoxal. Comparison with L-aspartate aminotransferase. *Photochemistry and Photobiology*.

[19] Arrio-Dupont M (1971). The effect of solvent on the fluorescence of Schiff bases of pyridoxal 5′ phosphate. *Biochemical and Biophysical Research Communications*.

[20] Lodha PH, Aitken SM (2011). Characterization of the side-chain hydroxyl moieties of residues Y56, Y111, Y238, Y338, and S339 as determinants of specificity in *E. coli* cystathionine *β*-lyase. *Biochemistry*.

[21] Pioselli B, Bettati S, Demidkina TV, Zakomirdina LN, Phillips RS, Mozzarelli A (2004). Tyrosine phenol-lyase and tryptophan indole-lyase encapsulated in wet nanoporous silica gels: selective stabilization of tertiary conformations. *Protein Science*.

[22] Schnackerz KD, Tai CH, Simmons JW, Jacobson TM, Rao GS, Cook PF (1995). Identification and spectral characterization of the external aldimine of the O-acetylserine sulfhydrylase reaction. *Biochemistry*.

[23] Lodha PH, Jaworski AF, Aitken SM (2010). Characterization of site-directed mutants of residues R58, R59, D116, W340 and R372 in the active site of *E. coli* cystathionine *β*-lyase. *Protein Science*.

[24] di Salvo ML, Florio R, Paiardini A, Vivoli M, D'Aguanno S, Contestabile R (2013). Alanine racemase from *Tolypocladium inflatum*: a key PLP-dependent enzyme in cyclosporin biosynthesis and a model of catalytic promiscuity. *Archives of Biochemistry and Biophysics*.

[25] Daum S, Tai CH, Cook PF (2003). Characterization of the S272A,D site-directed mutations of O-acetylserine sulfhydrylase: involvement of the pyridine ring in the *α*,*β*-elimination reaction. *Biochemistry*.

[26] Hunter GA, Ferreira GC (1999). Pre-steady-state reaction of 5-aminolevulinate synthase: evidence for a rate-determining product release. *The Journal of Biological Chemistry*.

[27] Krupka HI, Huber R, Holt SC, Clausen T (2000). Crystal structure of cystalysin from *Treponema denticola*: a pyridoxal 5′-phosphate-dependent protein acting as a haemolytic enzyme. *EMBO Journal*.

[28] Clausen T, Kaiser JT, Steegborn C, Huber R, Kessler D (2000). Crystal structure of the cystine C-S lyase from *Synechocystis*: stabilization of cysteine persulfide for FeS cluster biosynthesis. *Proceedings of the National Academy of Sciences of the United States of America*.

[29] Clausen T, Huber R, Laber B, Pohlenz H, Messerschmidt A (1996). Crystal structure of the pyridoxal-5′-phosphate dependent cystathionine *β*-lyase from *Escherichia coli* at 1.83 Å. *Journal of Molecular Biology*.

[30] Breitinger U, Clausen T, Ehlert S (2001). The three-dimensional structure of cystathionine *β*-lyase from Arabidopsis and its substrate specificity. *Plant Physiology*.

[31] Dwivedi CM, Ragin RC, Uren JR (1982). Cloning, purification, and characterization of *β*-cystathionase from *Escherichia coli*. *Biochemistry*.

[32] Droux M, Ravanel S, Douce R (1995). Methionine biosynthesis in higher plants. II. Purification and characterization of cystathionine *β*-lyase from spinach chloroplasts. *Archives of Biochemistry and Biophysics*.

[33] Lang T, Kessler D (1999). Evidence for cysteine persulfide as reaction product of L-cyst(e)ine C-S-lyase (C-DES) from *Synechocystis*: analyses using cystine analogues and recombinant C-DES. *The Journal of Biological Chemistry*.

[34] Nagasawa T, Ishii T, Kumagai H, Yamada H (1985). D-Cysteine desulfhydrase of *Escherichia coli*. Purification and characterization. *European Journal of Biochemistry*.

[35] Ravanel S, Job D, Douce R (1996). Purification and properties of cystathionine *β*-lyase from *Arabidopsis thaliana* overexpressed in *Escherichia coli*. *Biochemical Journal*.

[36] Gentry-Weeks CR, Spokes J, Thompson J (1995). *β*-cystathionase from *Bordetella avium*. Role(s) of lysine 214 and cysteine residues in activity cytotoxicity. *The Journal of Biological Chemistry*.

[37] Ejim LJ, D’Costa VM, Elowe NH, Loredo-Osti JC, Malo D, Wright GD (2004). Cystathionine *β*-lyase is important for virulence of *Salmonella enterica* serovar typhimurium. *Infection and Immunity*.

[38] Cellini B, Bertoldi M, Montioli R, Voltattorni CB (2005). Probing the role of Tyr 64 of *Treponema denticola* cystalysin by site-directed mutagenesis and kinetic studies. *Biochemistry*.

[39] Bertoldi M, Cellini B, D’Aguanno S, Voltattorni CB (2003). Lysine 238 is an essential residue for *α*,*β*-elimination catalyzed by *Treponema denticola* cystalysin. *The Journal of Biological Chemistry*.

[40] Kezuka Y, Yoshida Y, Nonaka T (2012). Structural insights into catalysis by *β*C-S lyase from *Streptococcus anginosus*. *Proteins*.

[41] Mehta PK, Hale TI, Christen P (1993). Aminotransferases: demonstration of homology and division into evolutionary subgroups. *European Journal of Biochemistry*.

[42] Milić D, Demidkina TV, Faleev NG, Matković-Čalogović D, Antson AA (2008). Insights into the catalytic mechanism of tyrosine phenol-lyase from X-ray structures of quinonoid intermediates. *The Journal of Biological Chemistry*.

